# The Role of Vitamins in the Pathogenesis of Asthma

**DOI:** 10.3390/ijms24108574

**Published:** 2023-05-10

**Authors:** Dominika Zajac, Piotr Wojciechowski

**Affiliations:** Department of Respiration Physiology, Mossakowski Medical Research Institute, Polish Academy of Sciences, 02-106 Warszawa, Poland; pwojciechowski@imdik.pan.pl

**Keywords:** asthma, vitamins, risk of developing asthma, airway hyperreactivity, airway inflammation, oxidative stress, airway remodeling, maternal intake, pregnancy

## Abstract

Vitamins play a crucial role in the proper functioning of organisms. Disturbances of their levels, seen as deficiency or excess, enhance the development of various diseases, including those of the cardiovascular, immune, or respiratory systems. The present paper aims to summarize the role of vitamins in one of the most common diseases of the respiratory system, asthma. This narrative review describes the influence of vitamins on asthma and its main symptoms such as bronchial hyperreactivity, airway inflammation, oxidative stress, and airway remodeling, as well as the correlation between vitamin intake and levels and the risk of asthma in both pre- and postnatal life.

## 1. Introduction

Asthma is a heterogeneous inflammatory disease of the airways. Currently, about 300 million people worldwide suffer from this disease [1]. Its symptoms comprise tightness of the chest, wheezing, dyspnea, and cough, all of which are accompanied by reversible airway obstruction and persistent inflammation of the airways. At the local level, the asthma-related changes include disturbances in the balance of Th1/Th2 pro- and anti-inflammatory cytokines, an increased cellular influx into the bronchi, chronic oxidative stress, and airway hyperreactivity. Within time or in the case of poor control of the disease, remodeling of small airways may occur [2,3]. Uncontrolled asthma leads not only to impairments of every-day-life such as a decreased physical condition, chronic fatigue, sleep problems, and obesity, but also to dangerous complications including obstructive sleep apnea (OSA), respiratory failure, chronic obstructive pulmonary diseases (COPD), pneumonia, or the life-threatening status asthmaticus. There is no single well-defined cause of asthma, but rather a number of risk factors, among which allergies, environmental pollution and irritants, genetic factors, excessive hygienic measures in childhood, frequent respiratory infections in early life, prenatal exposure to certain medications, and obesity are the most important. Asthma exacerbations can be induced by many factors such as allergen or irritant exposure, the use of specific medication, increased physical activity, and cold or viral infections [4]. A healthy lifestyle with appropriate physical activity and a balanced diet covering the proper levels of micro-, macro-nutrients, and vitamins may improve the control of the disease and alleviate its symptoms. The role of micronutrients in asthma has recently been summarized by Zajac [5], and this paper, as a kind of continuation, aims to describe the role of vitamins and their deficiencies in the pathogenesis of asthma.

Reports on the role of vitamins in asthma can be divided into three main groups. The first one includes studies on the general nutritional habits seen as a healthy lifestyle, including the Mediterranean diet and an increased consumption of fruits and vegetables, fish, and unsaturated oils. These reports rely mostly on diet diaries, specially designed questionnaires, or other recordings of food intake during a certain period. On the basis of these data, the authors conclude on the vitamin levels taken by a subject. In some of them, supplements from other sources are taken into account.

The second group of analyses limits its data to certain groups of vitamins, such as antioxidant vitamins (A, C, and E) or group B vitamins. The data are based either on surveys with detailed questions on the subpopulation of compounds tested or on direct observations of the subjects supplementing the given vitamin(s).

The third type of research focuses on direct supplementation (or depletion—in animal studies) of the given vitamin to the subject.

A proper sum-up of the role of vitamins in asthma is challenging for several reasons, all of them due to the character of the published studies. Some studies have concentrated on general healthy lifestyles and proper nutrition and described the relationship between the given diet (Mediterranean diet or avoidance of processed foods, etc.) and asthma. Others, in turn, have rather described the importance of a class of vitamins (water vs. fat soluble, antioxidant vitamins, etc.) in the prevention, risk of development, or treatment of asthma. Further articles have dealt with the role of one chosen vitamin, its deficiency or excess in the pathogenesis of asthma, and the possibility of its supplementation in the treatment of the disease. This review is, to our knowledge, one of very few taking together the influence of all vitamins, and not only general nutrition or each vitamin alone, on asthma. Specifically, for the first time, we focus not on asthma as a disease, but on its features, such as impaired lung function, airway hyperreactivity, airway inflammation, chronic oxidative stress, mucus oversecretion, or airway remodeling, together with viral infections being the main trigger of its exacerbations. Another problem we address in the present review is the role of maternal vitamin intake in asthma development in children, mentioning also the importance of gut microbiota. In consequence, the present paper aims to summarize all these findings in order to clarify the influence of vitamins on asthma and its main manifestations. The articles were searched in Pubmed and Google Scholar using the following terms (“vitamin A”, “vitamin B1”, “vitamin B2”, “vitamin B3”, “vitamin B5”, “vitamin B6”, “vitamin B7”, “vitamin B9”, “vitamin B12”, “vitamin C”, “vitamin D”, “vitamin E”, “vitamin K”, “thiamine”, “riboflavin”, “niacin”, “nicotinamide”, “nicotinamide riboside”, “pantothenic acid”, “pyridoxine”, “pyridoxal 5′-phosphate”, “biotin”, “folic acid”, “folate”, “tetrahydrofolic acid”, “cobalamin”, “ascorbic acid”, “1,25-dihydroxyvitamin D”, “tocopherol”, and “tocotrienol”) and (“asthma”, “risk of asthma”, maternal risk of asthma”), (“airway hyperreactivity”, “lung function”, “oxidative stress”, “cellular influx”, “mucus”, and “airway remodeling”) within the years of 2000–2023. Older articles were taken under consideration only if they provided information that could not be found elsewhere in newer publications and was fundamental to the subject.

## 2. Healthy Lifestyle, Nutrition, and Asthma

In general, a diet rich in antioxidants and anti-oxidant vitamins (vit. A, C, and E) decreases oxidative stress and lowers the risk of inflammation. Interestingly, in various studies, no association between anti-oxidant vitamins and asthma could be found [6,7], most likely due to dissimilarities in the basic diets in different regions, the potential fortification of food or supplementation from different sources (herbal or natural medicines, OTC (over the counter), or prescribed drugs), and the possibility of non-controlled use of vitamin supplements. For example, different results of studies are obtained regarding the influence of vitamin E on the asthma outcomes in Europe and America, where different oils are used for every-day cooking. The amounts of different isoforms of vitamin E (of the pro-inflammatory gamma-Tocopherol (γTPh) and the anti-inflammatory alpha-Tocopherol (αTPh)) vary between these oils. Therefore, the regular intake of the vitamin E isoforms present in the respective oils may interfere with supplemented vitamin E (see below). Moreover, the overconsumption of highly vitamin-A- and D-containing food, such as cod liver, may increase the risk of adult-onset asthma [8].

Another concern about vitamins and their involvement in the pathogenesis of asthma is the problem of deficiencies, which can be related to malnutrition, lifestyle, or place of residence. Only in 2014, about a billion people, especially those with respiratory disorders, suffered from a vitamin D deficiency, making it a global problem that could, in fact, be easily eliminated [9]. The risk factors of developing both asthma and a vitamin D deficiency are shared and overlap, and include air pollution, smoking, the use of certain medications, a sedentary lifestyle with low or absent outdoor activity, metabolic disorders, and others [10,11,12,13,14,15]. Other cultural habits, including traditional or weather-dependent clothes covering the body to a smaller or higher degree, the types of activities undertaken during free time, and the consumption of processed food or beverages as additional meals or snacks may influence vitamin levels and facilitate the occurrence of their deficiencies.

## 3. General Characteristics of the Vitamins

### 3.1. Vitamin A and Carotenoids

Retinoic acid (RA), the main active form of vitamin A, is essential for pre- and postnatal development, eye sight, reproduction, and immune function [16]. It is stored and later activated in the liver from retinol. The retinoic acid pathway (retinoic acid and receptors) regulates more than 500 genes, including those involved in immunity [17].

Regarding asthma, vitamin A promotes the normal development of the lungs, the differentiation and growth of respiratory epithelial cells [18], and enhances the proliferation and survival of T cells [19,20,21]. Together with other carotenoids, vitamin A is a ROS (reactive oxygen species) scavenger and has, in consequence, antioxidant and immunomodulatory properties. As such, they have been shown to decrease ROS (including superoxide anion and hydrogen peroxide) levels and suppress pro-inflammatory cytokines (explained in detail below) [22].

The form of the administration of retinoids is important. A liposomal formulation of all-trans retinoic acid (ATRA) aggravates asthma outcomes and exacerbates allergic inflammatory responses in OVA mice, while other forms of dosage alleviate them [23]. The exact mechanisms underlying this phenomenon are not known. Similarly, Reifen et al. [24] described that the aerosol administration of vitamin A has, contrary to other ways, no influence on asthma outcomes in children. An excess of vitamin A increases the risk of a child developing asthma when the compound is administered in a water-based formulation instead of an oil-based one [25]. Once again, the reasons of such difference have not been established yet.

In the literature, not only is vitamin A taken under consideration, but also other carotenoids, such as carotenes or even lycopene, which, however, is not transformed into vitamin A. The general consumption of carotenoid-rich food leads to a lower risk of asthma [26], better asthma control, and lung function, probably due to the antioxidant properties of this class of compounds [27,28].

### 3.2. Vitamin B Group

The vitamins of the B family are a group of eight water-soluble vitamins that are involved in various metabolic processes, including the formation of red blood cells. Moreover, they are cofactors (or their direct precursors) of enzymes. Only some of them participate in the processes related to asthma. Up until now, there have been no reports about a possible link between this disease and vitamins B1, B2, B5, and B7. Later in the text, a vitamin of this group is only mentioned when research data about its levels, intake, or influence on a feature of asthma are available.

The term vitamin B3 covers three substances, niacin, nicotinamide, and nicotinamide riboside, which are all included in the structure of nicotinamide adenine dinucleotide (NAD) and its phosphorylated derivative, nicotinamide adenine dinucleotide phosphate (NADPH), key coenzymes involved in metabolism and energy production. Their role and the role of NADPH-dependent oxidase in inflammation, asthma, and other respiratory diseases have been summarized by Harijith [29] and Chang [30]. In summary, NADPH-dependent oxidases participate in the stimulation of mucus production, neutrophil recruitment, the induction of ciliary dysfunction, and airway smooth muscle hypercontractivity. The role of nicotinamide as a molecule itself in asthma is not known, even if its levels are higher in asthmatics.

Vitamin B6, pyridoxine, and its active form, pyridoxal 5′-phosphate, is a coenzyme of some crucial enzymes. The compound is involved in amino acid metabolism, the synthesis of neurotransmitters including dopamine and serotonin, glucose and lipid metabolism, and hemoglobin synthesis. It enhances magnesium absorption. Vitamin B6 affects cell-mediated immunity; its administration improves immune responses. The role of vitamin B6 in these processes has been presented by Ueland et al. [31].

Vitamin B9, including folic acid, folate, and related compounds such as tetrahydrofolic acid participate in metabolic processes at the cellular level and act as methyl donors, a group of substances transporting single-carbon groups between bioactive substances. They participate in the methylation of DNA, its synthesis, and modification. Folates are needed in the activation process of cobalamin (vitamin B12), which, in turn, transforms homocysteine into methionine; thus, they are essential in the folate–methionine cycle and for methyl transport in cells [32]. Folate deficiencies in prenatal life are related to neural tube malformations and congenital heart defects. To prevent such issues, food fortification with folates is recommended worldwide, together with folic acid supplementation prior to and during pregnancy [33].

Vitamin B12, cobalamin, and its derivatives are another group of methyl donors that participate in the methylation and transformation of homocysteine to methionine. They play roles in the formation of blood cells, DNA synthesis, the synthesis of myelin, and the correct functioning of the nervous system [32]. Although vitamin B12 can be seen as an immunomodulator [34], its direct role in respiratory diseases is not known [5].

### 3.3. Vitamin C

Vitamin C (ascorbic acid) is a water-soluble compound that plays a role in tissue repair, collagen production, the proper activity of various enzymes, and the absorption of certain nutrients and minerals such as iron. Vitamin C is known for its anti-oxidant and anti-inflammatory properties [35,36]. It is believed to alleviate the symptoms of viral airway infections such as the common cold [37]. Moreover, smoking-induced airway irritation and cough are reduced by vitamin C supplementation or a vitamin-C-rich diet [38]. High levels of vitamin C are observed in the lungs, lung macrophages, and type II cells [39]. However, the supplementation of vitamin C does not change its levels in the lining fluid of the respiratory tract [40].

The most important challenge in research on the function of vitamin C with animal studies is that rats and mice have the ability to synthesize vitamin C on their own; thus, they are not prone to developing a vitamin C deficiency [35,41]. Hence, the majority of studies are performed on guinea pigs that are not capable of synthesizing this vitamin [42]. The data on the protective role of vitamin C in the respiratory system may vary between species. In animal models of ozone-induced asthma, the levels of vitamin C after exposure to the stimulus may either increase or decrease, depending on whether the animal of choice is mouse or guinea pig, respectively [41].

### 3.4. Vitamin D

Recently, increasing attention has been paid to vitamin D, its deficiency, and its role in immunity. The latter has been widely described by Kerley et al. [43,44] and its involvement in immunity has been discussed in excellent reviews by Chirumbolo et al. [45], Mora et al. [17], Hewison [46,47], Bikle [48], and Colotta et al. [49]. Mathyssen et al. [50] summarized the findings about its metabolism. One of the most important properties of vitamin D in asthma is its ability to inhibit inflammation by regulating the proliferation, migration, and cytokine release from various immune cells including mast cells, neutrophils, and eosinophils. Vitamin D has been shown to induce a decrease in the production of pro-inflammatory cytokines including IL-1β, IL-6, and TNFα, and an increase in the secretion of the anti-inflammatory IL-10 [43,44,45,46,47,48,49,50].

There are several forms of vitamin D, with vitamin D3 (cholecalciferol) and D2 (ergocalciferol) being the most important in mammalian and human health.

Vitamin D receptors have been found throughout the respiratory system, including epithelial smooth muscle cells [51], where the vitamin is converted into its active form (1, 25-dihydroxyvitamin D) [52,53]. Vitamin D is produced by immune cells, including macrophages, T, and B cells [54,55,56]. It has been found to play a role in mast cells, as described by Liu et al. [57]. In a vitamin-D-deficient medium, murine mast cells release more markers of their activation, including TNFα and histamine, than in a vitamin-D-sufficient medium, with this process being reversed by vitamin D administration.

One of the difficulties of asthma treatment and control is poor responses to inhaled corticosteroids (ICS) [3,4]. The correct level of vitamin D is crucial, as vitamin D downregulates the genes responsible for steroid resistance and thus enhances steroid responsiveness [12], especially in severe asthma [58,59], which, in turn, enables better asthma control. Interestingly, in the case of a deficiency, the supplementation of vitamin D can restore the proper response to ICS [60,61], probably directly at the levels of lymphocytes and monocytes [60].

In addition, it is interesting that better protection by vitamin D is observed when the vitamin is administered in an oil-based formula rather than in a water-based one [25].

### 3.5. Vitamin E

At least eight different forms can be called ‘vitamin E’: four tocopherols (alpha, beta, gamma, and delta TPh) and four tocotrienols (alpha, beta, gamma, and delta TT), all slightly different in mode of action [62]. The most widely described and of the greatest clinical significance are alpha and gamma tocopherol (αTPh and γTPh). According to various studies, these two isoforms have opposite properties [63] and the lack of an exact notice about the isoform used in the respective study leads to inconclusive results on the influence of vitamin E on animal and human health and disease. Additionally, not only does the isoform influence the results [62], but so does the model (allergic vs. non-allergic asthma) and species (mice vs. rats) used [64]. Another problem related to inconclusive results is the solvent. Some oils contain high amounts of γTPh (such as soy bean, canola (rapeseed), peanuts, or sesame). Others, in turn, do not contain it or contain it in small amounts (e.g., sunflower, safflower, olive, or grape seed) [62]. Therefore, the results may vary because of the type of oil used in the examined population (North and South America vs. Asia vs. Europe). At the same time, a higher prevalence of asthma has been observed in regions where oils with a high γTPh content are consumed [62]. αTPh supplementation in a γTPh-rich oil can block its beneficial actions and thus has no positive effect on asthma [65]. What is important in the context of animal experimentation is that regular mouse chow does not contain γTPh [66]. Abdala-Valenzia et al. have published a detailed list of studies involving α and γTPh in lung diseases, including the isoform and species used [67]. Nevertheless, there are papers, summarized by Jiang et al. [68], that have discussed the beneficial effects of γTPh, which would be similar to those of αTPh. Since mammalians cannot convert the isoforms of vitamin E [67], there is no possibility of transforming αTPh to γTPh, even in a time of high demand.

Vitamin E deficiency occurs commonly in malnutrition or absorption diseases, as vitamin E is fat soluble [69]. Furthermore, disorders of transport proteins such as the αTPh transport protein lead to vitamin E deficiency, as shown in knock-out mice by Lim et al. [70].

Unlike other vitamins, plasma TPh levels correlate with lung levels [62]. However, a lower αTPh level is often observed in airway lining fluid but not in plasma together with vitamin C deficiency [67].

Another bioactive form of vitamin E is tocotrienols (TT), with γTT being the most bioactive form [71,72]. Its potency against asthma symptoms is comparable with that of prednisolone, a high-efficiency corticosteroid [73]. Furthermore, tocotrienols are stronger antioxidants than other isoforms of vitamin E [74].

### 3.6. Vitamin K

There is only a limited number of publications linking vitamin K and lung diseases. Nevertheless, vitamin K and its derivatives and analogues might be useful in asthma [75], as their administration has ameliorated the asthma outcomes in 90% of mild, 86% of moderate, and 72% of severe adult asthmatics after one year of treatment [76]. Vitamin K reduces inflammation via the inhibition of NLRP3 inflammasome [77]. Moreover, vitamin K analogues decrease the levels of pro-inflammatory cytokines released in vitro by stimulating PBMC cells in allergic and non-allergic subjects [78]. Animal studies have shown that dietary vitamin K supplementation decreased the inflammatory markers in lipopolysaccharide (LPS)-induced lung injury [79], which, together with the antioxidant properties of vitamin K [80], suggests a possible use of this class of compounds in other inflammatory states of the respiratory system.

## 4. Vitamins and the Risk of Asthma

A healthy lifestyle, a balanced diet with the proper consumption of vitamins, accompanied by moderate physical activity, decreases the risk of various diseases, including lung diseases such as asthma, and diminishes their risk factors such as obesity. Low vitamin levels can be related to decreased intake and absorption or increased demand. Lower levels usually lead to the impairment of homeostasis and thus to an increased risk of developing diseases, including asthma, allergy, and general inflammation [81]. Not only can a deficiency be a risk factor for diseases, but an overload or excessive intake can contribute to the development of pathologies, as antioxidant vitamins, under certain conditions, can act as antioxidants.

The most important is the question of if there is a well-defined diet that could reduce the risk of asthma, allergy, and other respiratory diseases. This diet or lifestyle should cover the entire needs of the organism towards micro- and macronutrient vitamins, proteins, fats, and carbohydrates in correct proportions. It seems that the Mediterranean diet, composed of a high intake of fruits, vegetables, cereals, and olive oil, with moderate quantities of white meat, fish, and dairy products, and a low intake of sugar and red meat, could be one of the best nutritional patterns. Some meta-analyses [82] have indicated its protective role against the development of childhood asthma, but, interestingly, not allergy. In others, however, no association between the adherence to this diet during pregnancy and childhood asthma could be found [83]. Nevertheless, as described below, the correct levels of vitamins may lower the risk of developing asthma.

### 4.1. Vitamin A

According to Lee et al. [84], vitamin A deficiency in children, accompanied by exposure to tobacco smoke, increases the risk of asthma. At the same time, Huang et al. [85] found a negative association between vitamin A intake and the risk of asthma in Taiwanese asthmatic adolescents, while Checkley et al. [86] observed that vitamin A supplementation in early life in regions with chronic vitamin A deficiencies was not associated with a lower asthma risk; Green and Fascetti [87] also observed vitamin A supplementation to be helpful in asthma. Articles on the relationship between asthma and vitamin A have recently been lately summarized and analyzed by Hu et al. [88], who state that, according to the existing studies, there is no relationship between the risk of asthma and serum vitamin A levels.

### 4.2. Vitamin B Family

There are no reports linking asthma and vitamins B1, B3, B5, and B7. Where riboflavin (vitamin B2) is concerned, its intake correlates with a lower risk of developing an allergy [89]. There have been some attempts to link vitamin B12 levels and the risk of asthma, but the papers describing this were withdrawn and no further studies have been published.

There are nearly no data on the relationship between pyridoxine (vitamin B6) intake and asthma. There has been an attempt to determine whether vitamin B6 supplementation could ameliorate steroid-dependent asthma, but it did not show any benefits of such treatment [90]. On the other hand, Collipp et al. described that B6 supplementation ameliorated asthma outcomes and decreased the use of bronchodilators, including steroids [91]. Newer reports are missing.

The relationship between folate (vitamin B9) intake and asthma is not well established. The consumption of folate-rich green vegetables is part of a healthy lifestyle and thus reduces the risk of asthma [92]. It seems that a higher folate consumption from different sources is a kind of protection in postnatal life against the disease [93].

### 4.3. Vitamin C

In general, the consumption of fruits, vegetables, and other foods containing high amounts of antioxidants, including antioxidant vitamins such as vitamin C, diminishes the risk of asthma, wheezing, and other respiratory disorders [94,95]. There is a strong correlation between vitamin C intake and its plasma levels [96]. Asthmatic patients generally have a lower intake of vitamin C than other subjects [97,98]. Therefore, vitamin C intake is negatively associated with the risks of asthma [85] and airway hyperresponsiveness [99], and, at the same time, positively with lung function [100,101]. However, the use of vitamin C as a therapeutic agent alone in asthma is not recommended [102], since, according to Troisi et al. [103], there is no direct protective effect of vitamin C supplementation.

### 4.4. Vitamin D

Vitamin D deficiency is a risk factor for developing asthma [104,105] and an increased susceptibility to allergic sensitization [106]. Simultaneously, vitamin D and its receptors are necessary for the development of an inflammatory response—vitamin-D-receptor-deficient mice do not develop experimental asthma [107,108].

### 4.5. Vitamin E

The isoforms of vitamin E seem to play essential roles in immunity, allergy, and asthma. An intake of αTPh-low and γTPh-high oils or supplements increases the risk of asthma and decreases lung function, whereas αTPh-high and γTPh-low oils or supplements have the opposite action. Moreover, γTPh levels, but not those of αTPh, are associated with allergic sensitization [109]. Supplementation with an αTPh-high γTPh-low composition decreased inflammatory markers and airway hyperreactivity (AHR) [110] and improved lung function parameters, such as forced expiratory volume in 1 s (FEV1), in well-controlled allergic asthmatics [111].

There are doubts about if vitamin E supplementation is beneficial towards asthma and the risk of developing the disease. On the one hand, Pearson et al. [112] could not find any advantages of this treatment, including its influence on asthma outcomes or ICS demand, and nor could Rubin et al. [113]. On the other hand, a low vitamin E intake aggravates the asthma severity in asthmatics [97,114] and further decreases their lung function [101]. A higher consumption of vitamin-E-containing supplements decreased the airway hyperresponsiveness to ozone and other irritants in asthmatic [115] and healthy [116,117] subjects and increased the lung function in patients suffering from exercise-induced asthma [118]. At the same time, according to Cook-Mills et al. [119] and Fogarty et al. [120], vitamin E supplementation seems to play a protective role in the development of asthma and allergy.

## 5. Maternal Intake of Vitamins and Asthma in the Offspring

In recent decades, the importance of prenatal factors in the health statuses of children has been emphasized. The period of fetal development seems to have a major impact on later life, and prenatal exposure to medications, pollutants, or oxidative stress may contribute to various metabolic, neurological, or respiratory disorders, including asthma [121]. Expecting mothers are highly encouraged to follow a healthy lifestyle, avoid medications without medical advice whenever possible, quit smoking, and take pregnancy-dedicated vitamin formulations, etc., in order to avoid the possible risks of mineral and vitamin deficiencies and ensure the correct development of their child. One of the compounds of such preventive supplementation is folic acid, which is applied to prevent neural tube malformations. However, as stated below, it is not without any further concerns regarding the development of lung diseases.

### 5.1. Vitamin A

It is not clear if maternal vitamin A supplementation influences the development of asthma in a child. Maslova et al. [122], Checkley et al. [86,123], and Stelmach et al. [124] could not find any relationship between maternal vitamin A supplementation and asthma in children at different age points (one to seven years old).

However, in a study by Parr et al. [125], based on questionnaires about food and supplement intake in Norwegian pregnant women, it has been found that an excess administration of vitamin A, of up to 2.5 times above the official recommendations of 10,000 IU per day [126], increased the risk of asthma in school children, probably due to the generally vitamin-rich diet in Western countries. The fact that a high vitamin A intake can increase the risk of asthma in a child was recently stated in the meta-analysis of Hu et al. [88].

### 5.2. Vitamin B Group

Miyake et al. [127] could not find any link between the maternal consumption of this vitamin and the risks of asthma, wheeze, or eczema in a child. As pyridoxine (vitamin B6) is supplied in nearly all formulations containing magnesium ions, in order to increase their availability, it is very difficult to establish a relationship between its maternal intake or deficiency and asthma in human studies.

The scarce data on the influence of maternal intake and prenatal exposure to folates on the risks for children remain contradictory. One of the reasons for these ambiguous results may be the source of folic acid in the diet of the mother. Folate can be delivered through a diet rich in vegetables, or through folate-fortified food (flour) and the supplements recommended for pregnant women. The other is the short duration of the studies, based on self-reported questionnaires without evaluating the real folate status of the patient. Another reason might be the time frame of the supplementation (whole pregnancy, first/second/third trimester, etc.), as well as the time of follow-up or the end-point of the study, and the age of the offspring at the time of diagnosis [128].

Some researchers have found that maternal folate intake increases the risk of developing asthma and other atopic diseases in a child [129,130,131,132,133,134]. Others, in turn, have shown that folate intake (from food and supplements) only in the late stages of pregnancy increases this risk [135], and only in atopic women [136]. In contrast, according to Veeranki et al. [137], folate supplementation from the first trimester increases this risk, while the introduction of supplementation afterward has no impact on asthma in a child. Other researchers have concluded the same, and could not find relationships between maternal vitamin B9 intake and asthma in a child around the world [138,139,140,141,142,143]. However, as summarized by Sharma and Litonjua [32] and Blatter et al. [139], methyl donors, including folate and vitamin B12, are only one of many prenatal risk factors contributing to the development of asthma or atopy in offspring.

There are no data about a link between B12 levels or the intake of a mother and asthma in a child. In human studies, only van der Valk et al. [144] has stated that there is no association between B12 cord blood levels at birth and asthma or eczema at the age of 6.

### 5.3. Vitamin C

In addition to the obvious relationship between the healthy lifestyle of a mother and the health of a newborn, maternal vitamin C supplementation normalizes smoking-related changes in DNA methylation and thus decreases the incidence of wheeze and asthma in children at any age, despite a smoking history of the mother [145,146], as has been shown inter alia in a long-term study for the same group of children at 3 and 12 months and 5 years [147,148,149]. Moreover, vitamin C supplementation improves the lung function of children [150]. It is not clear whether vitamin C can counteract the possible folate-related changes in DNA methylation.

### 5.4. Vitamin D

Similarly to other vitamins, especially anti-oxidant ones, the correct levels and appropriate intake of vitamin D promote the correct development of offspring. A low maternal intake of vitamin D increases the risk of wheeze and asthma but not of allergy (eczema or hay fever) at the various age points of a child [151,152,153,154,155,156,157], and a high vitamin D intake protects from these two diseases [125,153,155,158,159,160,161,162]. Furthermore, it strengthens the immune responses to stimuli [163]. In mice, a higher maternal vitamin D intake leads to increased IL-10 and decreased IL-3 releases from the airway epithelial cells of the offspring [164]. The protective role of vitamin D is related to its influence on early lung development, including alveolar development and surfactant production [165,166]. Therefore, vitamin D supplementation during the entire pregnancy improves a child’s lung function [167]. However, others have not found an association between maternal intake, umbilical cord blood levels, and the risk of asthma [167,168,169,170,171].

### 5.5. Vitamin E

Most researchers agree that vitamin E supplementation during pregnancy has a positive effect on the risks of asthma, wheeze [172], and the lung function of a child [173], whereas a vitamin E deficiency increases these risks [151,152,173,174,175,176]. However, Stelmach et al. [124], Greenough et al. [177], and Maslova et al. [122] could not find any association between cord blood levels or maternal intake and the risk of asthma in offspring. This may be related to the fact that an increased intake of γTPh-containing oils increases the risk of asthma [62], and further supplementation with αTPh or a mixture of vitamin E isoforms counteracts the negative influence of γTPh. This has been shown in mouse studies, where αTPh supplementation during gestation decreased and γTPh increased the susceptibility to asthma in the pups [178,179].

### 5.6. Vitamin K

Even if vitamin K is believed to play an anti-inflammatory role in asthma and other lung diseases (see above), Maslova et al. [122] found that increased maternal vitamin K intake was associated with an increased risk of asthma in a 7-year-old child. Still, this is the only paper dealing with this problem, and the subject needs further investigation.

### 5.7. Vitamins and the Gut Microbiota

Another important issue is the influence of the maternal intake of vitamins and other nutrients on a mother and child’s gut microbiota (described in detail by Gao et al. [180], Gray et al. [181], and Alsharairi [182]). Healthy maternal microbiota is one of the factors decreasing the risk of asthma [180,183], probably by the induction of Treg lymphocytes in the fetal lung. This, in turn, occurs through the proper production of short-chain fatty acids by bacteria [181]. Maternal nutrition and possibly bacterial supplementation seem to be crucial for the proper functioning of the gut flora. Although the role of vitamins in these processes is still under investigation, Padilha et al. [184] found positive correlations between vitamin C intake and the *Staphylococcus* levels in human milk. This is important, as allergic children have lower levels of these bacteria in their feces than healthy subjects [184].

## 6. Vitamins, Vitamin Levels and Asthma Features

### 6.1. Vitamin Levels in Asthma

Correct levels of vitamins are strongly related to the general well-being of patients, and chronic diseases can be both a cause and a consequence of deficiencies. Increased oxidative stress, as observed in asthma and other inflammatory diseases, leads to a drop in the concentration of antioxidant vitamins such as vitamin C or E. Lower levels of these vitamins due to insufficient supply or extensive demand may enhance oxidative stress and its consequences. Usually, asthma is accompanied by decreased levels of vitamins. However, even if links between vitamin levels and asthma and its symptoms are established, it is still doubtful which of them, asthma or the vitamin deficiencies, is the cause and which is the consequence. Another interesting but still unexplained problem is the lower intake of vitamins themselves or foods containing high quantities of vitamins by asthmatic patients, as has been observed for vitamin C (see below). The levels of vitamins in asthmatic patients, together with the relationship between maternal intake and the risk of a child developing asthma, are presented in Table 1.

### 6.2. Main Features of Asthma

#### 6.2.1. Airway Hyperreactivity and Lung Function in Asthma

Airway hyperreactivity (AHR) is one of the key features of asthma. It is related to the extensive constriction of the bronchial tree in response to inhaled stimuli, leading to the narrowing of the airways, an increase in air flow resistance, and a reduced air flow, described often as decline in FEV1, thus leading to impaired lung function [185,186]. AHR is strongly related to airway inflammation, both acute and chronic character [187]. Acute airway inflammation, leading to a variable form of AHR, is usually reversed by inhaled glucocorticosteroids (ICS). Chronic airway inflammation, which results in the fixed form of AHR, is related to structural changes in the airways, the so-called airway remodeling (see below) [185,188]. The influence of vitamins on AHR is primarily related to the suppression of airway inflammation and the preclusion of early-stage structural changes in the airways (airway remodeling). Airway narrowing as a process leads to alterations in lung function, worsening over the course of asthma [186].

Decreased respiratory parameters occur in most patients with asthma [186] and are aggravated within the course of the disease [189]. However, this decline can be reduced in the course of proper asthma control with ICS and smoking cessation [190].

#### 6.2.2. Oxidative Stress

One of the outcomes and, at the same time, causes of asthma is oxidative stress, which cannot be balanced with the natural defense mechanisms of organisms such as anti-oxidant enzymes, including superoxide dismutase (SOD) and glutathione peroxidase (GPx) [191]. Oxidative stress, especially if chronic, leads to the development of inflammation via the promotion of T cell differentiation towards Th2 phenotypes [192,193]. Therefore, the supplementation of anti-oxidant vitamins, such as vitamin C, may prevent the development of inflammation. Most vitamins share anti-oxidant and anti-inflammatory properties, and in some cases, it is difficult to distinguish them considering their overall beneficial actions against asthma. Riedl and Nel [194] have published a detailed description of the role of oxidative stress in asthma.

#### 6.2.3. Airway Inflammation and Cellular Influx into the Airways

The key feature of asthma is chronic inflammation of the airways. Depending on the phenotype of the disease, it can be classified into allergic/nonallergic and eosinophilic/non-eosinophilic inflammation, etc. [2,195]. Not only is the abovementioned chronic oxidative stress one of the causes of airway inflammation in asthma, but so is an imbalance of the Th1/Th2 responses shifted towards the Th2 ones, leading to an increased production and release of pro-inflammatory cytokines and a reduction in anti-inflammatory cytokines by activated inflammatory cells [196]. Nevertheless, the immunological aspect of asthma is very complex and varies between the phenotypes of the disease [3].

#### 6.2.4. Mucus Production and Structural Changes within the Airways (Airway Remodeling)

Another feature of asthma is the impairment of the bronchial epithelium, leading to mucus hyperproduction and hypersecretion and impaired mucociliary clearance [197,198], which, in turn, leads to a further decline in lung function and promotes chronic inflammation [199].

Structural changes in the bronchial tree (airway remodeling) are a complication of severe asthma. They lead to structural alterations in the airways and persistent limitations of air flow. There are at least three components leading to airway remodeling: first, disturbances at the level of the airway smooth muscles, second, disturbances of collagen deposition leading to fibrosis, and third, disturbances at the level of the airway epithelium. All these causes of remodeling have been discussed in reviews by Camoretti-Mercado and Lockey [200], Fang et al. [201], Papa et al. [202], and Boulet [203]. Disturbances in mucus production and secretion are reversible, whereas changes leading to airway remodeling seem to be permanent.

### 6.3. Vitamin A

#### 6.3.1. Vitamin A Levels in Asthma

There is evidence about a link between vitamin A deficiency and asthma. According to Luo et al. [204], Al Senaidy [205], and other authors [97,114,206,207,208,209,210,211], asthmatics, independent of age, have a lower intake and levels of vitamin A and general carotenoids, even without showing any signs of deficiency. This has been confirmed in a broad meta-analysis by Hu et al. [88]. It appears that the levels and consumption of this group of substances are negatively correlated with the severity of asthma [207] and positively with lung function and quality of life [208,212,213,214,215,216,217]. As described by Gilliland et al. [101], poor lung function is associated with, among others, a low intake of antioxidant vitamins, including vitamin A. In Brazilian children, Ferreira et al. [218] showed lower retinol levels in asthmatic and asthmatic obese children, particularly with moderate and severe asthma. This is an important observation, as asthma and obesity share and are risk factors for each other [219].

In turn, Okuda et al. [132] in Japan and Schock [220] in Ireland could not find any association between asthma and serum carotenoid levels. Others have shown that an overdose of vitamin A and D due to an increased consumption of cod liver oil was associated with an increased incidence of adult-onset asthma [8]. The same has been observed by Laerum et al. [221]. On the other hand, Allen et al. [114] showed a negative association between vitamin A intake and asthma.

Interestingly, as studied by Misso et al. [98] and Rerksuppalhol [222], there was no difference in carotenoid intake nor levels between asthmatics and controls in Australia and Thailand, respectively.

Important in the context of the direct action of carotenoids in the airways is that their levels in sputum are lower than those in plasma, but the two values appear to be correlated [223].

#### 6.3.2. Vitamin A, Lung Function, and Airway Hyperresponsiveness

As retinoic acid (RA), the active form of vitamin A, and its signaling pathways play a role in smooth muscle differentiation during airway formation, vitamin A deficiency during the prenatal period leads to pulmonary defects and structural changes in the airway smooth muscles (ASM) of mice [123,224,225,226], which can contribute to non-inflammatory airway hyperreactivity [227]. Importantly, these alterations in muscle structure can be reversed by vitamin A supplementation [123]. The prenatal lung is affected by disturbances of vitamin A levels. Retinoic acid (RA) signaling is crucial for the regulation of adult smooth muscles. RA-deficient smooth muscles are hypertrophic, hypercontractile, and pro-fibrobiotic via the increased activation of the transforming growth factor beta (TGFβ) signaling pathway. The inhibition of this signaling path prevents changes related to RA deficiency. A short pharmacological interruption of the RA signaling in adult mice leads to a decrease in airway function, an increase in airway smooth muscles (ASM) thickness, and collagen disposition, resulting in ASM hypertrophy and airway remodeling [228,229].

The role of retinoid X receptors (RXR) in asthma and immunity has been widely described by Fujii et al. [230], Denburg et al. [231], and Spiegl et al. [232]. In summary, the administration of a partial agonist of RXR during an OVA challenge in the OVA asthma model decreased nearly all the symptoms of the disease, including airway hyperreactivity, cellular influx into the airways, and the levels of pro-inflammatory cytokines in the BALF, together with goblet cell metaplasia. At the same time, the authors of these publications pointed out that the full RXR agonist may have numerous side effects. Kanagaratham et al. [233] performed similar studies, in which fenretinide, a vitamin A derivative, was administered to mice prior to an ovalbumin (OVA) sensitization and challenge. The compound abolished nearby all the features of asthma, including the AA/DHA ratio (the ratio of pro- (arachidonic) to anti-inflammatory (docosahexaenoic) fatty acids; a marker of the control of inflammation), oxidative stress (malondialdehyde (MDA) and nitrotyrosine levels), cellular influx into the airways, the levels of pro-inflammatory cytokines, and AHR, most of them to control levels (of healthy animals). This shows the potential of vitamin A and its derivatives in prevention and control. The same was observed by Sahamoto et al. [234] for an ATRA (all-trans retinoic acid) administration during OVA sensitization in mice, where a decrease in AHR, together with an alleviation of other asthma symptoms, could be observed. Dietary vitamin-A-deficient adult rats had a higher AHR [227], however, this was without a change in the airway wall thickness. The authors concluded that vitamin A may be essential in the regulation of airway hyperresponsiveness and, at the same time, in the maintenance of normal bronchial epithelium. Parallelly, Parr et al. [125] stated that an excess of vitamin A can accumulate in lungs, and, together with retinoid metabolites, cause asthma-like symptoms. This was confirmed by Schuster et al. [235], who found that extensive vitamin A intake increases the risk and severity of asthma in OVA mice, whereas a slight vitamin A deficiency decreases AHR. In terms of lung function, the serum levels of vitamin A and carotenoids are positively associated with parameters such as forced expiratory volume in 1 s (FEV1) or forced vital capacity (FVC) [236].

#### 6.3.3. Vitamin A and Oxidative Stress

Vitamin A is accepted to be an antioxidant vitamin that reduces oxidative stress [22]. At the same time, it is still not clear whether vitamin A levels are correlated with those of oxidative stress markers in the airway. Bishopp et al. [237] could not find any relationship between vitamin A levels and exhaled nitric oxide (FeNO) and other oxidative stress markers among adults with uncontrolled asthma. At the cellular level, vitamin A seems to protect airway epithelium from oxidative stress-related injury [238,239]. In humans, a lycopene formulation in subjects with exercise-induced asthma improved lung function, probably through an antioxidant action [240]. Arora et al. [207] pointed out that decreased levels of vitamin A might be due to an increased use of this anti-oxidant during the chronic oxidative stress present in asthma. Wood and Gibson [241] found that asthmatics with airway hyperreactivity have lower beta-carotene levels and, at the same time, impaired (lower) plasma antioxidant potential.

#### 6.3.4. Vitamin A, Inflammation and Cellular Influx into the Airways

Vitamin A modulates the immune system [17], mostly by the hampering of the allergy-related Th2 and pro-inflammatory Th17 pathways [19]. The administration of RA during OVA or a house dust mite (HDM) challenge in asthma and allergic rhinitis in mice decreased both inflammatory AHR and rhinitis symptoms by inhibiting Th2 / Th17 differentiation and Th2 responses [242,243,244]. According to Maruya [245], vitamin A deficiency lowers IgA levels. An interesting problem has been shown by Tian et al. [246]. Another inflammatory state, neonatal pneumonia, decreased vitamin A levels. Vitamin A supplementation in the post-infection period increased the levels of Th1 and decreased those of Th2 cytokines after OVA sensitization, together with reductions in cellular influx into the airways and airway hyperreactivity. This shows that a vitamin A supplementation after infection inhibits the progression of asthma. Additionally, as mentioned by Gozzi-Silva [81], the correct vitamin A levels may even protect against infections.

One of the forms of vitamin A, ATRA, also promotes anti-inflammatory responses. As stated by Wu et al. [244], it decreases Th2/Th17 differentiation and reduces the total cell, especially eosinophil, influx into the airways in pretreatment or during an OVA sensitization and challenge in mice. Similar results have been shown by Takamura et al. [247], Sakamoto et al. [234], and Fang et al. [248] in rats. In contrast, vitamin-A-deficient OVA-sensitized mice show signs of increased inflammation [229], mediated by an activation of the Th2 cytokines, and increased neutrophil and eosinophil influx into the airways [229]. This can be reversed by RA administration [249,250].

Another example can be lycopene, a carotenoid that is not transformed into vitamin A. The administration of it to mice during OVA sensitization abolishes most asthma features, including AHR, cellular influx into the airways, and levels of Th2 cytokines [251,252], which shows that lycopene supplementation can be protective against asthma.

#### 6.3.5. Vitamin A, Mucus Production, and Airway Remodeling

It seems that vitamin A with its isoforms, as reported by Aggarwal et al. [253] for ATRA, plays an important role in the maintenance of the normal mucociliary phenotype. A supplementation with ATRA during OVA sensitization decreased goblet cell metaplasia and, as it is reversed by the administration of antagonists of retinoic acid receptors, is involved in the regulation of proper mucus production [253].

To date, there have been no reports on the influence of vitamin A on airway remodeling. Since the entire retinoic acid signaling pathway seems to be dysregulated [254] and the administration of ATRA inhibited the migration of airway smooth muscle cells in vitro [255], it is possible that vitamin A supplementation reduces the abnormal migration of airway smooth muscle cells within the bronchi of asthmatic subjects. Still, this needs to be investigated.

### 6.4. Vitamin B Group

#### 6.4.1. Vitamin B Group Levels in Asthma

Both Husemoen [256] and Heinrich [257] failed to find any association between B2 intake and allergy or atopy. However, Comhair et al. [258] found higher levels of nicotinamide (vitamin B3) in the plasma of asthmatics, probably due to an increased consumption of NAD+ in the course of immunologic activation in asthma. A similar result was shown by Liu et al. [259], where asthmatics, especially those suffering from non-eosinophilic asthma, had higher levels of nicotinamide, which was positively correlated with the frequency of severe asthma exacerbation.

There are no differences between asthmatic and non-asthmatic subjects in pyridoxine (vitamin B6) levels. However, asthmatics had lower concentrations of its active, phosphorylated form, pyridoxal-5′-phosphate [260]. Furthermore, the use of theophylline, one of the drugs used in asthma treatment, decreased plasma vitamin B6 in the plasma [261].

Folate (vitamin B9) levels are lower in asthmatic children [262,263] and adults [264], regardless of diet. Lower folate levels lead to a higher prevalence of asthma and higher rate of severe exacerbations [262,265], together with a higher risk of atopy [266]. Cord blood folate levels did not have an association with asthma [144].

Until now, no correlations between B12 levels or intake and asthma outcomes have been found [36,256,265,267].

#### 6.4.2. Vitamins of the B Group, Lung Function and Airway Hyperresponsiveness

There are no data about the vitamins of the B group and lung function or AHR in asthma. Only higher folate levels are associated with better lung function [268]. At the same time, a lower folate level has no impact on the lung function in asthmatic children [266,269].

#### 6.4.3. Vitamins of the B Group and Oxidative Stress

There are no data on the influence of B vitamins on oxidative stress. Only Lin et al. [269] have pointed out that the beneficial effect of folate on asthma symptoms in postnatal life can be related to the antioxidant properties of the compound by reducing homocysteine levels.

#### 6.4.4. Vitamins of the B Group, Inflammation and Cellular Influx into the Airways

There is only one report, dated fifty years ago, on the influence of nicotinamide (vitamin B3) on the features of asthma by Bekier et al. [270] in a guinea pig OVA model of asthma. Nicotinamide supplementation during sensitization decreased mast cell degranulation, the histamine release from these mast cells, and bronchial spasm.

Among the other vitamins of the B group, only folate is described in the context of asthma and the inflammation of the airways. There is no association between folate plasma levels and airway inflammation in both asthmatic and non-asthmatic children [262,265,267]. At the same time, as described in animal studies by Wu et al. [271], correct folate levels participate in maintaining Th cell balance, as folate-deficient mice showed impaired Th cell maturation. Furthermore, too high folate levels may increase the risk of sensitization, and thus airway inflammation, in postnatal life [272]. Iscan et al. [273] described in their study that prenatal folate supplementation in mice, followed by postnatal OVA sensitization, led to increased airway inflammation and higher allergic responses to OVA, an effect that was even more pronounced after a longer folate administration. Similar results have been observed by Hollingsworth et al. [274], who found that maternal supplementation with methyl donors, including folates, led to genetic changes in the offspring, resulting in increased airway inflammation and hyperreactivity, higher IgE levels, and airway remodeling. These changes were shown to be heritable, which leads to the conclusion that dietary factors can lead to a heritable risk of allergic diseases and a vulnerability to airway inflammations.

#### 6.4.5. Vitamins of the B Group, Mucus Production, and Airway Remodeling

İscan et al. [273] described in their study that prenatal folate supplementation, followed by a postnatal OVA sensitization, led to increased airway inflammation and a higher allergic response to OVA, which may lead to structural changes in the airways. Furthermore, fibrosis and an increased proliferation of epithelial and subepithelial smooth muscles, together with fibrotic changes, have been observed. Yet, the role of any vitamin of this group or its deficiency in this asthma manifestation has to be investigated.

### 6.5. Vitamin C

#### 6.5.1. Vitamin C Levels in Asthma

Not only does a low vitamin C intake occur in asthma, but first of all, a low plasma level of the vitamin is observed both in children [211,275] and adults [85,96,98], and is more pronounced in the severe form of the disease [275]. Moreover, low vitamin C levels are associated with an increased risk and prevalence of asthma [98,113,276]. At the same time, a low plasma vitamin C level is often associated with low levels of antioxidant enzymes, such as SOD and glutathione peroxidase (GPx) [211], and these levels decrease in the BALF shortly after exposure to irritants [277]. Nevertheless, in other studies [220,278,279], no relationship between vitamin C levels or intake and the incidence of asthma or lung function [97] has been found.

#### 6.5.2. Vitamin C, Lung Function and Airway Hyperresponsiveness

As asthmatics have both lower vitamin C levels and intake, the supplementation of this vitamin may ameliorate the course of asthma by lowering the number of asthma attacks (exacerbations), the asthma symptom score, the ICS need [280], and decreasing respiratory symptoms such as sore throat or cough in exercise- and cold-induced asthma after stimulation [37,39]. Nonetheless, such supplementation has to be prolonged, since a short administration of the vitamin has no influence on the above mentioned outcomes [281].

One of the most important symptoms of asthma is airway hyperresponsiveness (AHR) to various stimuli. According to Soutar et al. [99], a deficiency in vitamin C leads to an increased AHR. Accordingly, vitamin C supplementation decreases AHR to methacholine, histamine, sulfur dioxide, or physical effort in various forms of asthma, including ozone- and exercise-related asthma [115,282]. This has also been shown for ozone-induced asthma in highly polluted big cities around the world, where vitamin C supplementation alone, or in combination with other antioxidant (pro)vitamins, such as beta-carotene and vitamin E, decreased AHR, as summarized by Fogarty [120]. These findings have also been shown in animal models, both in mice and in guinea pigs, where vitamin C supplementation, both general and during OVA sensitization, strongly reduced AHR [283].

Additionally, lung function is positively affected by vitamin C supplementation. Although Nadi et al. [284] could not find any influence of single or chronic vitamin C supplementation on the respiratory parameters in asthmatics, Ochs-Balcom et al. [214] have shown an increase in lung function in asthmatic subjects. So did acute and chronic vitamin C supplementation in exercise-induced asthma where it lowers the drop in respiratory parameters after physical effort [282,285,286].

#### 6.5.3. Vitamin C and Oxidative Stress

The only known mechanism of the action of vitamin C in oxidative stress in asthma is the ability to scavenge the free radicals that are produced in response to oxidative stress, but also released during uncontrolled physical activity in exercise-induced asthma. Thus, this decreases the levels of oxidative stress markers [39,287].

#### 6.5.4. Vitamin C, Inflammation, and Cellular Influx into the Airways

Like many other antioxidant substances, vitamin C also plays a role in immune defense and has anti-inflammatory properties. Vitamin C deficiency down-regulates the proper immune response to inflammatory stimuli and its supplementation up-regulates it, as mentioned above for common airway inflammation and infections.

Supplementation with vitamin C reduces inflammatory markers in the general population. It lowers CRP levels [288] and thereby reduces the severity of pneumonia [289]. It also increases white blood cell counts in adult asthmatics [284]. Simultaneously, vitamin C deficiency increases necrotic lung cell damage and prostaglandin levels after an in vitro H_2_O_2_ treatment and therefore inflammation in guinea pigs. Animal studies have shown that vitamin C supplementation reduces anti-OVA antibodies and IL-4 levels in an OVA rat model of asthma, along with a decrease in the markers of oxidative stress, such as MDA [39].

In OVA mice, i.p., the administration of vitamin C decreased airway cell influx during a sensitization and challenge [283]. Megadoses of vitamin C strongly inhibited the Th2 responses [290], eosinophilia, and neutrophilia in OVA mice and guinea pigs, respectively [35]. As a result, vitamin C was shown to modulate the Th1/Th2 responses towards Th1 [35] and thus act as an anti-inflammatory agent.

#### 6.5.5. Vitamin C, Mucus Production, and Airway Remodeling

It is not known whether vitamin C directly influences mucus production. Only one study has described the influence of vitamin C on airway remodeling. As reported by Kianian et al. [291], both administrations of vitamin C alone and in co-administration with vitamin D during an allergen sensitization and challenge decreases goblet cell hyperplasia and subepithelial fibrosis. However, vitamin C may play a role in the collagen deposition, as vitamin C is a cofactor of collagen production. Therefore, low vitamin C levels could lead to impairments in collagen formation and structure.

### 6.6. Vitamin D

#### 6.6.1. Vitamin D Levels in Asthma

Vitamin D levels correlate with the lung function in asthmatic [292,293,294] and healthy subjects [13,295]. Lower vitamin D levels are associated with chronic lung diseases [10] and an increased susceptibility to infections [208]. In this context, asthmatic patients show lower levels of vitamin D than healthy subjects, independent of age [12,208,292,293,296,297,298,299,299,300,301,302,303], and a more serious vitamin D deficiency correlates with asthma severity [208,302,304,305], lower asthma control, and ICS responsiveness [14,61,302,304,306,307,308,309,310]. Lower vitamin D levels increase the number of exacerbations of the disease [14,306,311,312], the risk of developing asthma and atopy [104,105], and the progression of the disease [313]. At the same time, asthmatic vitamin-D-deficient patients have higher ICS needs [58,308,310,314]. Vitamin D supplementation in subjects deficient in vitamin D decreases the numbers of asthma exacerbations and general asthma symptoms [315,316]. Moreover, it reduces the risk of developing wheeze in preterm children [317]. At the cellular level, a low vitamin D level aggravates inflammation via the promotion of M1 macrophage polarization. A supplementation of vitamin D restores the correct balance by promoting M2 macrophage polarization, leading to a kind of prevention of inflammation-related damage [318].

Contrary to the above mentioned papers, some researchers could not find any differences in the vitamin D levels between asthmatics and healthy controls [319,320] or associations between the levels of this vitamin and asthma [320,321,322,323]. Once again, this might be due to differences in the dietary habits, place of residence, age, and sun exposure of the analyzed populations.

#### 6.6.2. Vitamin D, Lung Function and Airway Hyperresponsiveness

Vitamin D administration has a positive effect on lung function and lung mechanics [13,15,295,324]. This vitamin is essential for the correct lung development and surfactant production in rats [160], but also in humans [325]. Vitamin D deficiency is associated with worse lung function [13,14,15], a higher risk of developing lung diseases, and a faster progression of these diseases [326,327]. Vitamin D supplementation improves lung function [310,328,329] and reduces the number of exacerbations [50,60,328,330,331], leading to better asthma control [50,332,333], a lower ICS need [331,333,334], and a higher quality of life, pronounced by decreased absences from school and work and increased activities of daily life [208,335]. Better outcomes of this vitamin D supplementation are observed in the pediatric population [60]. What highlights the importance of vitamin D in respiratory diseases is the observation that its level falls strongly during an asthma exacerbation [336]; thus, a vitamin D deficiency leads to an increase in AHR [309].

All these findings have been confirmed in animal studies. In vitamin-D-deficient OVA mice, vitamin D supplementation decreased AHR and IgE levels [337,338,339]. Furthermore, the transfer of vitamin-D-treated T cells to OVA mice prevented AHR and airway inflammation [340].

#### 6.6.3. Vitamin D and Oxidative Stress

Vitamin D is an anti-oxidant and promotes the antioxidant activity of other substances [341]. In the case of vitamin D deficiency, increases in oxidative stress and inflammation are observed [342]. Additionally, vitamin D protects mice from oxidative stress-related injury [343]. Adam-Bonci et al. [344] recently presented a very interesting study that not only summarized the latest findings on vitamin D in oxidative stress, but also showed the influence of short- and long-term vitamin D supplementations on the asthma symptoms in the serum, BALF, and lungs of OVA mice. Both treatments decreased the markers of oxidative stress, such as the oxidative stress index, total oxidative status, and lipid peroxidation, and increased the total antioxidant response. Additionally, vitamin D enhanced the activity of the enzymes involved in glutathione (GSH) production. Another observation was made by Lan et al. [341], where a vitamin D deficiency in human asthmatics increased ROS release and DNA damage, which, in turn, was reversed by vitamin supplementation.

#### 6.6.4. Vitamin D, Inflammation, and Cellular Influx into the Airways

Vitamin D has anti-inflammatory properties. It enhances Treg activity [60,345,346], downregulates pro-inflammatory cytokines [154,347,348,349,350,351], and up-regulates anti-inflammatory cytokines [61,347,350,351,352], both in vitro and in vivo. Furthermore, it decreases mast cell activation and histamine release [57,353]. Vitamin D is an immunomodulator [328] and its administration improves allergen immunotherapy [81,354]. As it has been observed, vitamin D shows antiviral actions in children [355,356,357,358,359] and its proper levels may protect against infections, one of the risk factors for asthma exacerbations [159,356,359,360] (see below).

In animal studies, vitamin-D-deficient asthmatic mice had increased eosinophilia, which was reversed by vitamin D [361]. OVA mice supplemented with vitamin D during sensitization and an allergen challenge had fewer symptoms of airway inflammation [343,362]. It seems that vitamin D stabilizes the balance between the Th1 and Th2 responses [363]. As was shown by Liu et al. [57], both healthy and OVA-sensitized mice on a vitamin-D-deficient diet had higher histamine and TNFα levels in their serum compared to animals receiving vitamin-D-sufficient or supplemented chow. This points out that vitamin D downregulates the mast cell activation increased in allergic sensitization.

The anti-inflammatory effects of vitamin D are based on three main inhibitory mechanisms: first, NFKB signaling; second, p38 MARK phosphorylation; and third, the activity of immune cells [364,365,366].

#### 6.6.5. Vitamin D, Mucus Production, and Airway Remodeling

There are only a few papers dealing with the problem of vitamin D and mucus. Lai et al. [362], Wang et al. [343], and Feng et al. [367] showed the relief of mucus disturbances in OVA mice with decreased goblet cell hyperplasia and mucus hypersecretion after vitamin D supplementation, suggesting that vitamin D may be a promising add-on therapy in asthma.

Vitamin D affects airway remodeling by decreasing smooth muscle proliferation [368,369]. Furthermore, it reduces the mass of ASM, epithelial collagen deposition, and goblet cell hyperplasia [362,368], all of which are increased in vitamin D deficiency [304]. It also downregulates the expression of the enzymes involved in airway remodeling [370,371,372,373]. In animal models, vitamin-D-deficient mice showed more pronounced airway remodeling, which was partially reversed by vitamin D supplementation [361]. At the same time, vitamin-D-supplemented OVA mice presented a reduced subepithelial collagen deposition [343,362]. Vitamin D receptors seem to be necessary for the maintenance of proper collagen synthesis, as vitamin D receptor knock-out animals had a higher collagen disposition than control subjects [374].

### 6.7. Vitamin E

#### 6.7.1. Vitamin E Levels in Asthma

Inconsistent observations about the levels of vitamin E and its isoforms and asthma are most likely due to the study region, the dietary and culinary habits of the population, and the form of supplementation, as described above. Most researchers state that lower levels or intake of αTPh are observed in asthmatics, independent of age and the severity of the disease [62,98,114,120,209,209,237,375,376,377], and can be corrected by supplementation [209]. At the same time, no differences in the levels of vitamin E among minor and adult populations [378], nor an association between vitamin E level and asthma risk [113,132,220,379] have been found. However, vitamin E levels and intake correlate with the lung function in stable [101,236,380,381] but not uncontrolled asthmatics [237]. On the contrary, de Luis et al. [97] could not find any relationship between vitamin E levels and lung function (measured as FEV1) in asthmatic and healthy subjects. At the same time, low vitamin E levels were correlated with markers of oxidative stress and inflammation, such as MDA or FeNO [209]. Once again, most studies miss the information about the composition of vitamin E.

#### 6.7.2. Vitamin E, Lung Function and Airway Hyperresponsiveness

In accordance with the theory of a beneficial action of αTPh and a detrimental one of γTPh, αTPh shows a positive correlation with lung function, whereas γTPh shows a negative one, both in asthmatic and non-asthmatic patients [62]. An αTPh administration decreased the AHR of atopic asthmatics and improved lung function in exercise-induced asthma [110,118]. In animal studies, both in OVA mice and rats, an αTPh and general vitamin E administration prior to various allergen challenges, including carbon and aluminum oxide nanotubes, decreased AHR [382,383,384]. Interestingly, this was observed also for γTPh during ozone exposure in OVA allergic animals [385]. At the same time, OVA mice on a vitamin-E-deficient diet showed more pronounced AHR [386].

#### 6.7.3. Vitamin E and Oxidative Stress

Both αTPh and γTPh have antioxidant properties [387,388]. As described by Patel et al. [389], αTPh acts only on ROS, whereas γTPh is active against both reactive oxygen and nitrogen species (ROS and RNS). Additionally, γTT decreases in an HDM mouse model of asthma ROS levels [73], but also, similarly to αTPh, induces the expression of the antioxidant enzymes SOD and GPx [73,390].

Vitamin E, independently of the isoform, decreases oxidative stress via at least two independent pathways. First, it directly scavenges free radicals and second, it increases the expression of NRF2 (Nuclear factor erythroid 2-related factor 2), a transcription factor regulating anti-oxidant and detoxifying enzymes [73]. A higher expression of NRF2 decreases oxidative-stress-induced lung injury [391], whereas a lower one leads to increased oxidative stress and inflammation and has been found in macrophages from asthmatic patients [392]. Similar results have been obtained in animal experiments [393] on OVA mice, where vitamin E supplementation restored the otherwise decreased NRF2 levels and thus partially reversed the symptoms of asthma.

In another asthma model related to oxidative stress, namely toluene-diisocyanate-induced (TDI), mice and rats exposed to oxidized graphene, single-walled carbon nanotubes, nano aluminum oxide, or other highly pro-oxidative substances, vitamin E administration decreased ROS [382,383,394,395].

Some researchers have pointed out that an antioxidant action of αTPh is not always observed. Okamoto et al. [384] found that αTPh administered prior to and during OVA sensitization had no influence on oxidative stress, even if it decreased other asthma symptoms.

Nevertheless, tocopherols can be ROS donors when they cannot be reduced by vitamin C during increased peroxidation processes [67]. This has been observed in patients exposed to high doses of vitamin E, when increased pro-oxidant activity was measured [396].

Vitamin E, together with vitamin A, C, and D, directly influences not only the oxidant–antioxidant balance and oxidative stress, but also the inflammation induced by oxidative stress, as described below.

#### 6.7.4. Vitamin E, Inflammation, and Cellular Influx into the Airways

The antagonistic effects of αTPh and γTPh on allergies and allergic inflammation have been described in detail [62,63,67,71,397,398]. γTPh seems to be a strong pro-inflammatory agent, about five–ten times stronger than the anti-inflammatory αTPh [62,71]. However, at physiological concentrations, γTPh does not directly modulate the expression of pro-inflammatory cytokines, but rather influences the regulation of endothelial cell signaling in leukocyte recruitment [66].

All the isoforms of vitamin E have immunomodulatory and anti-inflammatory properties [399,400] by inhibiting the release of pro-inflammatory cytokines, such as interleukin (IL)-1, IL-6, or tumor necrosis factor α (TNFα) [399,400]. A deficiency in vitamin E increases the levels of inflammatory markers, as reported by Talani et al. [386], where OVA mice on a diet deficient in vitamin E had elevated levels of F2-isoprostanes.

The anti-inflammatory action of αTPh has been proven in various studies, mainly in OVA mice and rats prior to and/or during an OVA sensitization and challenge. In all of these studies, αTPh reduced cellular influx into the airways, eosinophilia, and decreased the levels of pro-inflammatory cytokines such as IL-1, 4, 5, and 13, along with decreasing the levels of the IgE and NO metabolites in the lungs [383,384,401,402,403,404]. In healthy (non-asthmatic) mice, vitamin E supplementation decreased pro-inflammatory TNFα and increased the levels of the anti-inflammatory interferon γ (INFγ). These observations have been confirmed for the stimulated peripheral blood mononuclear cells (PBMC) from healthy humans [405]. Parallelly, αTPh and its metabolites decreased the activity of arachidonate 5-lipoxygenase (5-LOX) [406], leading to lower levels of pro-inflammatory leukotrienes. This is even more interesting, as vitamin E deficiency increases the expression and activity of 5-LOX [407].

The opposite effect of αTPh and γTPh has been described by Berdnikovs et al. [66] in an OVA mice model of asthma. αTPh decreased both cellular influx into the airways, airway inflammation, and AHR, whereas γTPh increased these parameters. The action of αTPh was antagonized by γTPh. Even if the two isoforms act contradictorily, the supplementation of a mixture of αTPh and γTPh, or even γTPh alone, can be useful in neutrophilic lung inflammation, including asthma, as according to several authors [385,401,408,409,410], as this type of inflammation is blocked by both tocopherols.

However, there are reports on a beneficial effect of γTPh. According to Suchankova et al. [411], αTPh supplementation in OVA rats had no major effect on asthma outcomes, whereas γTPh administration alleviated the majority of the symptoms of asthma, such as airway inflammation and mucus overproduction. This could be due to the solvent (soy oil), which contains large amounts of γTPh [62]. The same has been described by Wagner et al. [385], where oral γTPh supplementation decreased the inflammatory cell influx in OVA rats.

Another form of vitamin E, γTT, also shows anti-inflammatory properties. It decreased the inflammation regarded as increased AHR and pro-inflammatory cytokine levels in an HDM mouse model of asthma [73]. In vitro, it significantly decreased the levels of IL-1β, TNFα, IL-8, and NFκB [412,413]. It appears to be one of the most important pathways for the anti-inflammatory action of vitamin E in all its isoforms, occurring mainly via the inhibition of the NFκB pathway [414].

The administration of vitamin E before and after an OVA sensitization and challenge decreases not only oxidative stress and its consequences, including the development of airway inflammation (eosinophilia and the levels of pro-inflammatory cytokine levels such as IL-4, 5, and 13), but also AHR in OVA mice [384,403,415], especially in the presence of oxidative stress enhancers such as oxidized graphene, where vitamin E administration decreases Th2 responses [395].

Not only are the levels of the vitamin or a supplementation with one of its forms important, but so are the transport mechanisms within the body. Tocopherol transport proteins seem to be essential for the correct development of immune response to allergens. As presented by Lim et al. [70], tocopherol transport proteins in knock-out mice, when sensitized with OVA, had lower IgE levels and lower IL-5 and TNFα expression than sensitized wild mates. Consequently, vitamin E deficiency impairs immunity.

#### 6.7.5. Vitamin E, Mucus Production, and Airway Remodeling

Both α and γTPh supplementation, before and during a sensitization and allergen challenge in OVA mice and rats, decreased mucus production and goblet cell hyperplasia [383,384,385,395,402,403]. Furthermore, they restored the mitochondrial ultrastructure at the level of the bronchial epithelium [403]. At the same time, γTT, in an HDM mouse model of asthma, decreased epithelial cell hypertrophy and mucus production [73]. Hence, vitamin E seems to prevent or slow down the development of airway remodeling and altered mucus production.

A short summary of the influence of these vitamins on the main manifestations of asthma is presented in Table 2.

## 7. Vitamins and Asthma Exacerbations

The management of asthma control and prevention of its potentially dangerous exacerbations relies mostly on proper medication measurements and an avoidance of its triggers. The latter include, but are not limited to, viral and bacterial infections, allergens, drugs, or pollutants [4,416]. Decreased responses to allergens can result from immunotherapy and antiallergic treatments. Exposure to pollutants can rarely be totally avoided and, as most of them (ozone, particulate matters, and nitrogen and sulfur oxides, etc.) are pro-oxidants and thus pro-inflammatory, their influence on asthma exacerbations can be partially limited by an administration of anti-oxidant vitamins, which has been described above. In the case of drug-induced asthma, especially aspirin-induced asthma, therapeutics from other groups have to be used. Probably the most important cause of asthma exacerbations are viral infections [417], which can be partially avoided by vaccinations, if available. Most virus-induced asthma attacks are related to respiratory syncytial virus (RSV) and rhinovirus (RV) infections [418], and the exact mechanisms involved in the pathogenesis of this type of asthma are described in detail by Hansbro et al. [419] and Hershenson [420]. Other viral pathogens that promote asthma exacerbations are influenza viruses [421], but, surprisingly, not COVID-19, as ICS administered in regular asthma treatment seem to protect against the disease and asthmatic patients are less prone to COVID-19 [422,423,424,425].

A healthy lifestyle, including the appropriate consumption of fruits and vegetables, promotes the proper immune responses to pathogens and reduces the risk of asthma exacerbations [426]. The exact role of vitamins in immunity has been widely described in previous parts of the paper and a detailed description of their role in the protection against viral infections exceeds the scope of the present review. Nevertheless, vitamin deficiencies lead to an increased sensitivity to pollutants and allergens and a higher susceptibility to respiratory infections, while proper vitamin levels contribute to resistance to them. There are only a few reports directly linking vitamin deficiencies and virus-induced asthma exacerbations; thus, only findings on the role of vitamins in respiratory infections are herein presented in summary.

It is doubtful if vitamin A supplementation has any influence on respiratory tract infections (RTI). In meta-analyses, no [427] or only slight [428] effects on outcomes and the incidence of RTIs, including RSV, could be shown. For the B group, Vlieg-Boerstra [428] did not find any influence of folate or vitamin B12 supplementation on the incidence of RTIs; studies on other B vitamins are lacking. Despite the common beliefs about the beneficial action of vitamin C towards viral infections, there is little evidence that its administration would prevent an RTI [429]. According to Hemilä et al. [430] and Keya et al. [431], vitamin C administration does not prevent or reduce the number of RTIs, however, it may reduce the duration of the disease, especially in prone subjects. In contrast, a vitamin D supplementation, especially a regular one, reduces the incidence of acute RTIs [417,432,433,434,435,436,437]. Vitamin D deficiency at birth increases the risk of severe RTIs, including RSV, in infants [438,439]. Low levels of this vitamin throughout life are generally associated with a higher incidence of infections [440], which can be corrected by its supplementation [441]. Interestingly, only for vitamin D have studies been performed directly on asthmatic subjects. They have shown that, in this group of patients, vitamin D supplementation decreases the number of RTIs [442] and asthma exacerbations [315]. Where vitamin E is concerned, data are scare and contradictory. Some researchers have described that vitamin E supplementation decreases the incidence of common cold [433] or some RTIs [434]; others do not support these findings [428].

In short, it seems that only vitamin D supplementation decreases the risk of developing respiratory tract infections, which are a major trigger of asthma exacerbations. In fact, only single studies linking vitamin deficiencies or intake with the frequency of viral infections have involved asthmatic patients.

## 8. Summary

A healthy lifestyle and balanced diet with an appropriate amount of vegetables and fruits, which provide adequate levels of vitamins, can reduce the risk or severity of asthma. Asthmatic patients are deficient in almost all vitamins and a supplementation of any kind may ameliorate their state, primarily by reducing oxidative stress and inflammation, thus improving the symptoms of asthma and overall health. Thus it might be a good add-on therapy to the respective medications. This seems to be more important, as asthmatic patients tend to already have a lower intake of vitamins and vitamin-rich food than healthy subjects. Only in the case of vitamin E should attention be paid to a supplementation of the anti-inflammatory isoform (αTocopherol). Furthermore, the correct intake of vitamins from natural or pharmacological sources during pregnancy decreases the risk of developing asthma and wheeze in a child. Nevertheless, attention has to be paid to the correct doses of the respective vitamins. According to the latest recommendations from health authorities, too high a supplementation may even increase the risk of allergy or asthma in a child, as is seen in the cases of vitamin B9 (folate) and vitamin K. Moreover, a dietary background including every-day cooking habits may influence the outcomes of vitamin supplementation via food fortification (vitamins of the B group), the natural content of the isoforms of a vitamin in oils (vitamin E), or a high consumption of vitamin-rich food (vitamin A and D in cold-water fish).

**Figure 1:**
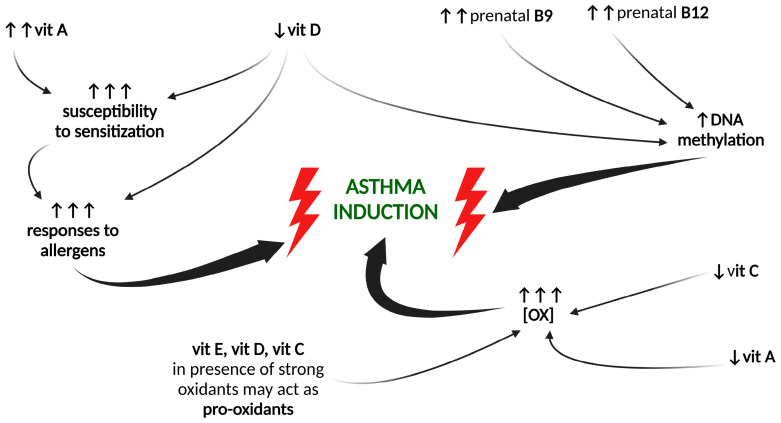
Relationship between vitamins and factors leading to asthma induction (for details, see text). ↓ low; ↑ high/excess; [ox] oxidative stress. Created with BioRender.com.

There are many factors that lead to asthma induction. Although the general risk factors for asthma are known, their detailed mechanisms and, in the context of this paper, the role of vitamins, are still under investigation. Prolonged exposure to pollutants (irritants, environmental, or air pollution) may lead to chronic inflammation, either by a direct irritation of the airways or by exposure to chronic oxidative stress. Moreover, mucus overproduction and consequent goblet cell hyperplasia and airway remodeling may occur. At this stage, epigenetic changes may come to light. Another risk factor are metabolic diseases including obesity and immunological disorders such as allergies of different origins. Additionally, prenatal exposure to certain medications and frequent respiratory infections can contribute to the development of asthma. It is quite impossible to avoid all these risk factors, however, as described above, the correct levels of vitamins may delay the onset of the disease or at least diminish its risk. In this context, low levels of the antioxidant vitamins C and A increase oxidative stress by lowering the antioxidant potential of the cells. Too high folate or cobalamin levels in prenatal life can lead to changes in DNA methylation, which, when natural repair mechanisms are impaired, can be chronic and irreversible, especially under vitamin C and D deficiencies. Too low vitamin D levels, but at the same time, chronic overdoses of vitamin A, may lead to an increased susceptibility to allergic sensitization and thus to higher responses to allergens. Additionally, vitamin D deficiency is predisposed not only to metabolic disorders, another risk factor for the development and progression of asthma, but also to respiratory tract infections, one of the triggers of asthma exacerbations. They are presented in Figure 1.

**Figure 2:**
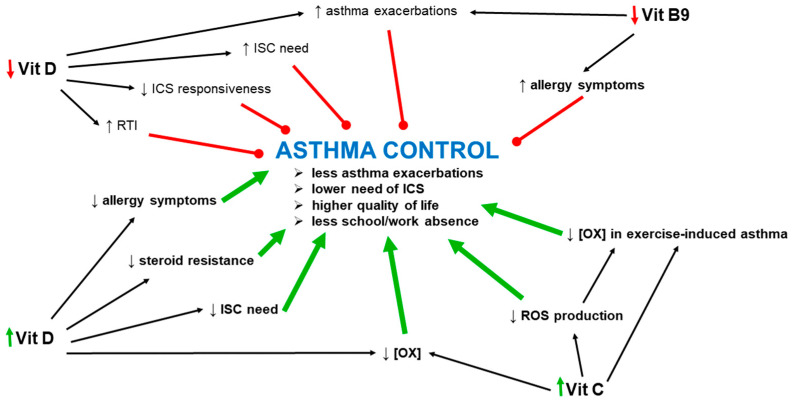
Factors contributing to asthma control (for details, see text). In green are factors improving asthma control, in red those worsening it. ↓ low/decrease; ↑ high/increase; RTI respiratory tract infections, ICS inhaled corticosteroids, [ox] oxidative stress.

Another important problem of asthma is its control (presented in Figure 2). Well-controlled asthma leads to a reduction in or suppression of its exacerbations, a lower need for inhaled glucocorticosteroids (ICS), a better quality of life, and better all-day activity. Low levels of vitamin D and folate increase the numbers of respiratory tract infections, asthma exacerbations, and allergy symptoms. Vitamin D deficiency lowers the ICS responsiveness and thus increases their need. In contrast, correct vitamin D levels lower these factors and reduce allergy symptoms and oxidative stress, and thus inflammation. Similarly, vitamin C, in adequate quantities, prevents ROS production and oxidative stress. Together, the correct levels of vitamins facilitate asthma control and reduce the risk of its induction.

**Figure 3:**
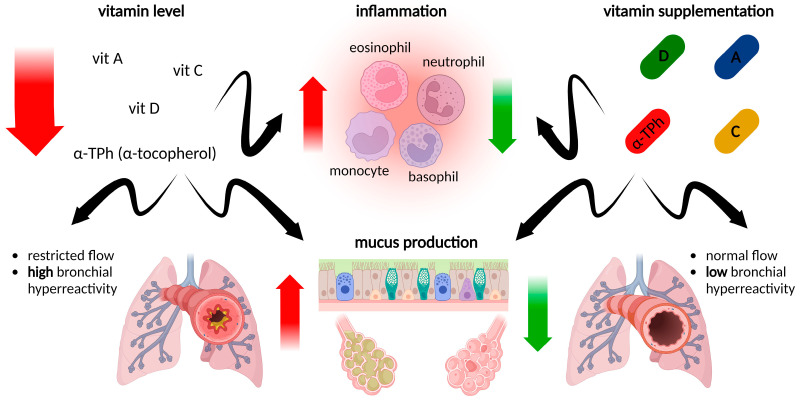
Effects of low vitamin level in asthma and outcomes of the vitamin supplementation (for details, see text). In green are factors improving asthma outcomes, in red those worsening it. ↓ low/decrease; ↑ high/increase; Created with BioRender.com.

The beneficial action of vitamin supplementation in asthma is mainly based on the influences of these vitamins on oxidative stress and inflammation. All the vitamins, other than the vitamins of the B group and vitamin K, decrease oxidative stress, either directly by free radical scavenging or indirectly by the promotion of antioxidant enzymes. Vitamins of the B group, others than vitamin B9 which at too high or too low levels may act pro-inflammatory, have no influence on inflammation. Vitamins A, C, D, and K suppress inflammation just as vitamin E does, as long as the beneficial anti-inflammatory effect of α-tocopherol predominates the pro-inflammatory activity of the γ isoform. The same applies to airway hyperreactivity as a consequence of inflammation. Interestingly, only vitamin D and E hamper airway remodeling and only the latter has some impact on mucus overproduction. Regular vitamin D supplementation reduces the number of respiratory infections, one of the most common causes of asthma exacerbation. Taken together, although vitamin A, C, D, and E have been well described in respiratory diseases, including asthma, the role of the vitamins of the B group still needs to be investigated, together with the mysterious vitamin K, which can be a new player in the field of respiratory disorders. A short summary of the findings is presented in Figure 3.

This is a narrative review, summarizing the most important research articles and pointing out the excellent reviews of other authors, without any detailed analyses of the findings or meta-analyses for each vitamin or all the vitamins together. These would be far beyond the scope of a single article. Therefore, the aim of the present paper was to mark the influence of vitamins on asthma features, together with the impact of their deficiencies on pathophysiology of the disease. More details on this broad subject can be found in the cited papers. Nevertheless, as underlined above, the final conclusions should be drawn with caution, as the intake and levels of vitamins or micronutrients strongly depend on the patient’s lifestyle, place of residence, or dietary and cultural habits. The estimations of vitamin intake may also differ depending on the type of study (diary-based, direct supplementation, and animal vs human studies, etc.). Thus, the obtained results may vary between the studied groups or populations. Other limitations of the presented papers include, in some cases, a lack of information about other data such as age, definition of asthma (including or excluding wheeze), smoking habits, the general health of the patients (comorbidities, obesity, or allergies), or level of indoor/outdoor activity. Animal studies may have less limitations, apart from a small number of subjects.

Another problem that is beyond the scope of this review is the genetic background of asthma. Epigenetic marks may influence gene expression and can be further modified by environmental factors. This opens up a new research field in the pathophysiology of various diseases, including asthma. Some aspects, including DNA methylation and histone modification, have been recently described by Sharma et al. [443].

Nevertheless, asthma is a very complex disease and many factors may lead to its induction, development, and progression. One of these factors is nutrients, including vitamins, whose role in the pathogenesis of asthma has been described in the present paper.

## Figures and Tables

**Table 1:** Vitamins, their levels in asthma, and the impact of maternal intake on asthma development in a child. (Data from animal and human studies, for details, see text).

Vitamin	Levels in Asthma	Increased Maternal Vitamin Intake and the Risk of Developing Asthma by the Child
A	↓	↑
B1, B2, B6, B7, B12	?	?
B3	↑	?
B9	↓	↑
C	↓	↓
D	↓	↓
E	↓	↓

↓—decrease; ↑—increase; ?—not known.

**Table 2:** Influence of vitamins on main asthma features. (Data combined from animal and human studies, for details, see text).

Vitamin	Lung Function	AHR	Oxidative Stress	Inflammation	Airway Remodeling
A	↑	↓	↓	↓	?
B	?	?	?	?	?
C	↑	↓	↓	↓	?
D	↑	↓	↓	↓	↓
E	↓↑ *	↓↑ *	↓	↓↑ *	↓
K	?	?	?	↓	?

* depending on the isoform. ↓—decrease; ↑—increase; ? not known.

## Data Availability

Not applicable.
